# Coordination of Macro Base Stations for 5G Network with User Clustering

**DOI:** 10.3390/s21165501

**Published:** 2021-08-16

**Authors:** Kun Li, Xiaomeng Ai, Jiakun Fang, Bo Zhou, Lingling Le, Jinyu Wen

**Affiliations:** State Key Laboratory of Advanced Electromagnetic Engineering and Technology, School of Electrical and Electronic Engineering, Huazhong University of Science and Technology, Wuhan 430074, China; likun_20@hust.edu.cn (K.L.); xiaomengai@hust.edu.cn (X.A.); zhoubo563@foxmail.com (B.Z.); lelele@hust.edu.cn (L.L.); jinyu.wen@hust.edu.cn (J.W.)

**Keywords:** 5G macro base station, energy management, BS sleeping, user clustering, Benders decomposition

## Abstract

With the increasing amounts of terminal equipment with higher requirements of communication quality in the emerging fifth generation mobile communication network (5G), the energy consumption of 5G base stations (BSs) is increasing significantly, which not only raises the operating expenses of telecom operators but also imposes a burden on the environment. To solve this problem, a two-step energy management method that coordinates 5G macro BSs for 5G networks with user clustering is proposed. The coordination among the communication equipment and the standard equipment in 5G macro BSs is developed to reduce both the energy consumption and the electricity costs. A novel user clustering method is proposed together with Benders decomposition to accelerate the solving process. Simulation results show that the proposed method is computationally efficient and can ensure near-optimal performance, effectively reducing the energy consumption and electricity costs compared with the conventional dispatching scheme.

## 1. Introduction

Fifth generation mobile communications technology (5G) is meant to deliver higher peak data speeds, ultra-low latency, increased reliability, massive network capacity, increased availability, and a more uniform experience to an increased number of users. Higher performance and improved efficiency enable new user experiences and connections to new industries. The upgrading of communication technology and equipment provides better services to users.

Despite the provision of better communication services to users, the main challenge faced by telecom operators is the increasing energy consumption of 5G equipment. According to data from China Mobile, the power consumption of a typical 5G macro BS exceeds 4 kW, which is approximately four times that of 4G. Considering the high deployment density of 5G BSs, the overall power consumption may reach 12 times the consumption of 4G networks [1]. The increase in the power consumption of 5G macro BS networks will directly lead to an increase in the operating expense (OPEX) of telecom operators. It is estimated that by 2025, the communications industry will consume 20% of the world’s electricity, and the electricity expenses will exceed 15% of the operating costs [2]. In addition, the carbon footprint of the global information and communication industry (ICT) accounts for 2% of global greenhouse gas emissions, with an annual growth rate of 6% [3]. To respond to the global call for green and low-carbon development, reduce the pressure on environmental protection, and reduce the OPEX of telecom operators, research on green communication techniques is necessary. During the past decade, most research on BS energy saving has been classified into two categories: hardware energy-saving technology (HEST) and software energy-saving technology (SEST).

HEST is proposed to improve the energy efficiency of 5G BSs with hardware designs, power source modification, and network architecture upgrades. At the BS level, research on the improvement of the energy efficiency of the power amplifiers (PAs) of active antenna units (AAUs) has been extensively studied [4,5,6]. Aiming to resolve the problem associated with the efficiency of the BS antenna changes with traffic load fluctuations, Popovic et al. summarized the power modulation method to improve the efficiency of PAs and indicated the future development direction and challenges associated with this technology [4]. A power amplifier design based on high-voltage GaN HEMT devices has been proposed that is able to transmit high-power signals and ensure the energy efficiency of the PA [5]. Ali et al. designed a linear complementary metal-oxide semiconductor (CMOS) power amplifier based on the use of an amplitude-modulation–phase-modulation (AM–PM) distortion compensation transformer to compensate for the distortion of high-power amplifiers in AM–PM. This improved the efficiency of the PA and ensured the equipment’s performance linearity [6]. In addition, studies on renewable energy for mobile network infrastructure represent a growing field. The application of PV panels and wind turbines in BSs helps to save electricity costs and reduce the carbon footprints of 5G networks [7,8,9,10,11]. Furthermore, some studies have proposed methods to deal with the uncertainties of renewable energy in economic dispatch, including the traditional cubic set modeling method [12], the extreme scenarios method [13], and the continuous-time modeling method [14]. At the network level, the application of cloud technology for the upgrading of the radio access network (RAN) architecture is another key technology that promotes energy conservation in 5G networks. With cloud computing techniques, computing tasks such as the baseband signal processing of BSs are transferred to a centralized data center. Ideally, all BSs are connected to a central server, which is referred to as a radio access network (cloud-RAN, C-RAN). In the C-RAN architecture, network deployment is more flexible, and the energy consumption of the network can be significantly reduced [15]. HEST is the basis for achieving energy savings in 5G networks and fundamentally improves the energy efficiency level of BSs. However, the realization of HEST relies on the evolution of the network architecture and the maturity of 5G key components. Therefore, the application of HESTs requires a certain period of time.

There is an obvious tidal effect in the wireless network business, presenting the characteristics of uneven traffic loads in time and space. SEST is dedicated to the improvement of the operation strategy of communication equipment, thus optimizing the resource allocation and shutting down redundant hardware by taking advantage of the distribution characteristics of traffic loads to realize the energy reduction of the 5G network [2]. SEST at the BS level includes symbol power off, slot power off, channel power off, and PA bias-voltage adjustment [16], which improve the energy efficiency of a single BS. A further reduction of 5G network energy consumption requires the cooperation of multiple BSs in the network. At the network level, the BS sleeping method was adopted in [17], and the energy efficiency of the cell networks subject to the average connection ratio (ACR) and average user rate constraints were optimized. Numerical results show that with the sleep strategy, the energy consumption of the cell network can be reduced by approximately 21%. Dutta et al. designed a distributed self-adaptive (SAS) algorithm for 5G BSs networks to improve energy and reduce their carbon footprint. Each BS independently and dynamically determines its operational state. Simulation results show that the proposed algorithm significantly increases energy savings [18]. Power allocation is another promising SEST used to reduce the energy consumption of 5G networks by allocating power resources to different users while meeting the users’ quality of service (QoS) [19]. Commonly used methods for determining power allocation include equal power allocation [20], the water-filling algorithm [21], branch and bound [22], and game theory [23]. Fang et al. studied the joint optimization of the subchannel allocation and power allocation of 5G heterogeneous networks, and the problem was formulated as a mixed-integer nonconvex optimization problem. Simulation results showed that the proposed algorithms can attain higher system energy efficiency [24]. Niu et al. investigated a cell zooming algorithm to adaptively adjust the cell size according to traffic load, user requirements, and channel conditions, greatly reducing the energy consumption and leading to green cellular networks [25,26,27]. Device-to-device (D2D) communication is an apt technology for proximity-based data sharing services and allows direct communication between users without a BS to reduce the transmission power consumption of BSs [28,29]. Moreover, the design expectations for 5G networks cannot be properly articulated by a single performance objective. Some research works have proposed multi-objective optimization frameworks for the planning and optimized operation of wireless communication networks [30,31]. However, there are two problems with the above SESTs. First, they only focus on the energy-saving problem of communication equipment in 5G BSs, while ignoring standard equipment [32] such as air conditioning (AC), backup batteries, and renewable generation units. In conventional operation strategies, backup batteries only provide an uninterruptible power supply (UPS) and are not dispatched most of the time [33]. The ACs in the cabinets are responsible for regulating the indoor temperature so that it does not exceed the upper and lower limits. However, the scheduling strategy of ACs can be improved to further reduce the energy consumption and electricity costs by making full use of the thermal inertia of the cabinets, because the indoor equipment has no comfort requirements. Second, as the number of BSs and users increases, wireless access networks become more complex, which makes it more difficult to solve the optimal dispatching scheme for 5G BSs.

To tackle the aforementioned challenges, this study proposes a dispatching scheme for a 5G macro BS network incorporating the optimal scheduling of standard equipment in the BSs. The main contributions of this study are as follows.

(1) A two-step energy management model for both communication equipment and standard equipment in the 5G macro BS network is proposed to reduce further the energy consumption and electricity costs. The cooperation among all BSs in the network is achieved through BS sleeping, user allocation, and power transmission methods.

(2) A user clustering method based on users’ geographic distribution is proposed to reduce the computational burden of the first-step problem. Benders decomposition is applied to accelerate the solution of the optimal scheduling of standard equipment.

The remainder of this paper is organized as follows. The two-step energy management model for both communication equipment and standard equipment is proposed in Section 2. Section 3 introduces the problem reformulation using the user clustering method and Benders decomposition algorithm. Section 4 presents and analyzes the simulation results, and the optimality and efficiency of the proposed model are verified, followed by the conclusions.

## 2. Energy Management Model of 5G Macro Base Station Network

**Figure 1:**
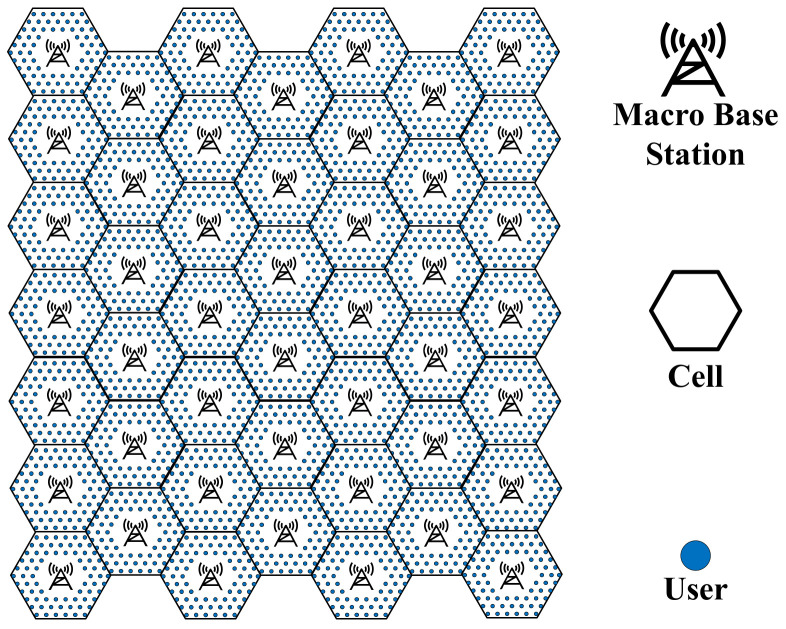
Schematic of a 5G macro BS network.

**Figure 2:**
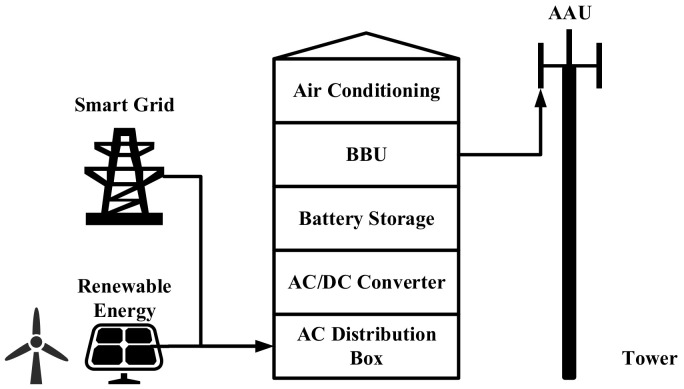
Diagram showing the structure of a 5G macro BS.

The 5G macro BS homogeneous network is shown in Figure 1. The main energy-consuming equipment in a macro BS include the communications equipment, an AC, a backup battery, and a renewable generation unit. The communication equipment consists of an active antenna unit (AAU) and a baseband unit (BBU), which are responsible for signal transmission and baseband signal processing, respectively. The coverage area of an active 5G macro BS is called a cell, which is a regular hexagonal area. The architecture of the 5G macro base station is shown in Figure 2. The AAU and renewable generation units are installed outside the base station cabinet. The remaining equipment is installed in the cabinet.

**Figure 3:**
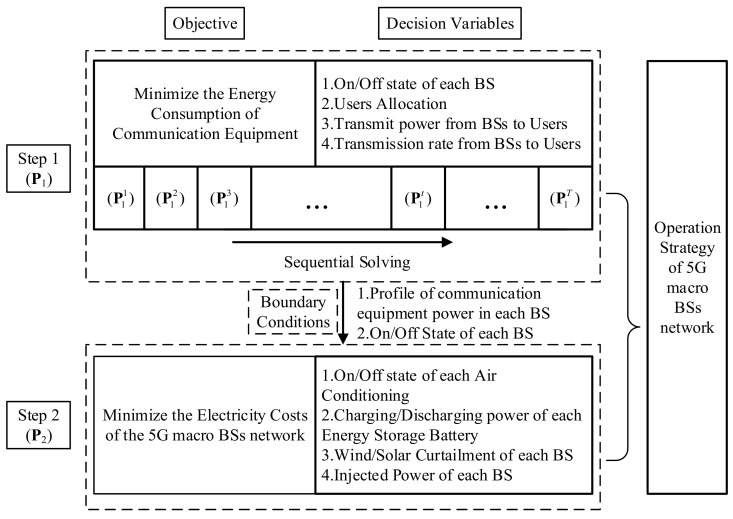
Framework of the energy management model of the 5G macro BS network.

The two-step energy management model for the 5G macro-BS network is illustrated in Figure 3. First, the energy consumptions of the AAU and BBU are minimized by optimizing the on/off state of the BSs and user allocation in the cellular network during each time period. Given the power profile and on/off state of each BS, the injected power of each BS, the on/off state of ACs, the charge/discharge power of backup batteries, and the power of renewable generation units during each time period are jointly optimized to achieve the goal of the economic operation of the 5G macro BS network.

### 2.1. Communication and Power Consumption Model of 5G Macro BS

The extensively used EARTH model was adopted to describe the power consumption of AAU and BBU [34], as shown in (1). The powers of BBU and AAU in BS *m* are a constant and a linear function of the transmitted signal power, respectively, when BS *m* is in active mode. The powers of BBU and AAU are two small constants when BS *m* is in sleep mode.

(1) $$\begin{array}{l} P_{com}^{m}=P_{BBU}^{m}+P_{AAU}^{m}\\ =\left\{\begin{array}{l} P_{BBU,Ac}^{m}+P_{AAU,Ac}^{m}\;\;,ActiveMode\\ P_{BBU,Sl}^{m}+P_{AAU,Sl}^{m}\;\;,SleepMode \end{array}\right.\\ =\left\{\begin{array}{l} P_{BBU,Ac}^{m,c}+\frac{1}{\delta_{PA}^{m}}P_{tr}^{m}+P_{AAU,Ac}^{m,c}\;\;,ActiveMode\\ P_{BBU,Sl}^{m,c}+P_{AAU,Sl}^{m,c}\;\;,SleepMode \end{array}\right. \end{array}$$

where $P_{com}^{m}$ denotes the power of communication equipment in BS *m*; $P_{BBU}^{m}$ and $P_{AAU}^{m}$ denote the powers of BBU and AAU in BS *m*, respectively; $P_{BBU,Ac}^{m}$ and $P_{AAU,Ac}^{m}$ denote the powers of BBU and AAU in BS *m* when BS *m* is in active mode, respectively; $P_{BBU,Sl}^{m}$ and $P_{AAU,Sl}^{m}$ denote the powers of BBU and AAU in BS *m* when BS *m* is in sleep mode, respectively; $P_{BBU,Ac}^{m,c}$ and $P_{AAU,Ac}^{m,c}$ denote the constant part of the powers of BBU and AAU in BS *m* when BS *m* is in active mode, respectively; $\delta^{PA}$ denotes the PA efficiency of the AAU in BS *m*; $P_{tr}^{m}$ denotes the total transmit power of AAU in BS *m*; and $P_{BBU,Sl}^{m,c}$ and $P_{AAU,Sl}^{m,c}$ denote the constant part of the powers of BBU and AAU in BS *m* when BS *m* is in sleep mode, respectively.

(2) $$P_{tr}^{m}=\sum_{u\in BSm}P_{tr}^{m,u}$$

where $P_{tr}^{m,u}$ denotes the transmit power allocated to user *u* by BS *m*, and ***BSm*** denotes the set of users connected with BS *m*.

We now present the downlink transmission model from BS *m* to user *u*. The AAU in BS *m* transmits electromagnetic waves to user *u* with power $P_{tr}^{m,u}$, which would attenuate in the air medium. The power of the electromagnetic waves received by user *u* is expressed as

(3) $$P_{re}^{m,u}=P_{tr}^{m,u}\cdot 10^{-\frac{A_{PL}\cdot d^{m,u}+B_{PL}}{10}}$$

where $d^{m,u_{t}}$ is the distance between BS *m* and user *u*; *A_PL_* and *B_PL_* are the path loss coefficients; and $P_{re}^{m,u}$ denotes the power of the electromagnetic waves received by user *u* from BS *m*. The power of the interference signal received by each user was considered constant in this study. According to Shannon’s equation, the data rate received by user *u* is expressed as

(4) $$L_{re}^{u}=B\cdot \log_{2}(1+\frac{P_{re}^{m,u}}{N_{0}})$$

where *B* is the channel bandwidth; *N*_0_ denotes the interference signal power received by each user; and $L_{re}^{u}$ denotes the data rate received by user *u*. According to (3) and (4), the transmit power of BS *m* to user *u* is given by (5) when the traffic load of user *u* is $L_{re}^{u}$.

(5) $$P_{tr}^{m,u}=N_{0}(2^{\frac{L_{re}^{u}}{B}}-1)\cdot 10^{\frac{A_{PL}\cdot d^{m,u}+B_{PL}}{10}}$$

### 2.2. Optimization Step 1: Energy Management of the 5G Communication Equipment

The proposed model assumes that the distribution of users and their traffic loads are known. The objective is to minimize the energy consumption of all AAUs and BBUs in the network during time period *t*, which is expressed as

(6) $$\min F_{1}^{t}=\min\sum_{m\in M}P_{com}^{m,t}\Delta t$$

where $P_{com}^{m,t}$ is the power consumption of the communication equipment (i.e., AAU and BBU); ***M*** denotes the set of all 5G macro BSs in the network; and Δt is the time step of one time period and is assumed to be equal to 15 min in this study.

BS sleeping and user allocation are adopted to reduce the energy consumption of 5G communication equipment in the network. During the periods of valley traffic load, some of the BSs could be switched to sleep mode, and the users served by these BSs could be transferred to adjacent BSs for service. According to Equation (1), although the transmitting power $P_{tr}^{m,t}$ needed by users would increase after transfer, the constant power parts $P_{AAU,Ac}^{m,c}$ and $P_{BBU,Ac}^{m,c}$ of these BSs are saved so that the total energy consumption of the communication equipment can be reduced.

The operation of communications equipment needs to meet the following constraints.

(1) Power consumption constraints of communications equipment:
  (7) $$P_{com}^{m,t}=P_{AAU}^{m,t}+P_{BBU}^{m,t}$$
  (8) $$P_{AAU}^{m,t}=\left\{\begin{array}{l} \frac{1}{\delta_{PA}^{m}}P_{tr}^{m,t}+P_{AAU,Ac}^{m,c},\;U_{bs}^{m,t}=1\\ P_{AAU,Sl}^{m,c}\;\;\;\;,\;U_{bs}^{m,t}=0 \end{array}\right.$$
  (9) $$P_{BBU}^{m,t}=\left\{\begin{array}{l} P_{BBU,Ac}^{m,c},\;U_{bs}^{m,t}=1\\ P_{BBU,Sl}^{m,c},U_{bs}^{m,t}=0 \end{array}\right.$$
  (10) $$U_{bs}^{m,t}\in \{0,1\}$$
  where $P_{com}^{m,t}$ denotes the power of communication equipment in BS *m*; $P_{AAU}^{m,t}$ and $P_{BBU}^{m,t}$ are the power consumptions of the AAU and BBU in BS *m*, respectively; $U_{bs}^{m,t}$ represents the working status of BS *m*; BS *m* is in sleep mode when $U_{bs}^{m,t}$ *=* 0 and active mode when $U_{bs}^{m,t}$ *=* 1; $\delta_{PA}^{m}$ is the PA efficiency of the AAU in BS *m*; *Pm,c AAU, Ac* and *Pm,c AAU, Sl* denote the constant parts of the power of AAU in BS *m* in the active and sleep modes, respectively; and *Pm,c BBU, Ac* and $P_{BBU,sl}^{m,c}$ are the constants of the power of BBU in BS *m* in the active and sleep modes, respectively.
(2) With the BS in sleep mode and the use of the user allocation method, each user in the network could be served by local or adjacent BSs. However, each user can only connect to one BS at a time, and its QoS requirements should be met. The user allocation constraints are as follows:
  (11) $$a_{t}^{m,u_{t}}\leq U_{bs}^{m,t},m\in N_{u_{t}}$$
  (12) $$\sum_{m\in N_{u_{t}}}a_{t}^{m,u_{t}}=1$$
  where $a_{t}^{m,u_{t}}$ denotes the connection relationship between BS *m* and user *u_t_*; user *u_t_* is not connected with BS *m* when $a_{t}^{m,u_{t}}=0$, while user *u_t_* is connected to BS *m* when $a_{t}^{m,u_{t}}=1$, and $N_{u_{t}}$ represents the set of adjacent BSs of user *u_t_*. Equation (11) indicates that user *u_t_* can only connect with BSs that are in active mode. Equation (12) indicates that user *u_t_* can only connect with one BS at a time.
(3) Maximum data transmission rate constraints of BSs:
  (13) $$L_{tr}^{m,t}=\sum_{u_{t}\in N_{m}}a_{t}^{m,u_{t}}L_{re}^{u_{t}}$$
  (14) $$L_{tr}^{m,t}\leq L_{tr}^{\max,m}$$
  where ***N****_m_* denotes the set of users in cell *m* and its adjacent cells; $L_{tr}^{m,t}$ denotes the total data processing rate of BS *m*; and $L_{tr}^{max,m}$ denotes the maximum data processing capacity of BBU in BS *m*.
(4) Maximum transmit power constraints of BSs:
  (15) $$P_{tr}^{m,t}=\sum_{u_{t}\in N_{m}}a_{t}^{m,u_{t}}P_{tr,t}^{m,u_{t}}$$
  (16) $$P_{tr}^{m,t}\leq P_{tr}^{\max,m}$$
  where $P_{tr}^{m,t}$ denotes the total transmit power of AAU in BS *m*; and $P_{tr}^{max,m}$ denotes the maximum transmit power of BS *m*. The maximum data transmission rate constraints (14) and the maximum transmit power constraints of BSs (16) ensure that all users’ QoS requirement can be satisfied.

Because Equations (8) and (9) are nonlinear constraints, the big-M method is applied to linearize them, as shown by Equations (17)–(24).

(17) $$P_{AAU}^{m,t}=P_{AAU,Ac}^{m,t}+P_{AAU,Sl}^{m,t}$$

(18) $$P_{AAU,Ac}^{m,t}\geq -M(1-U_{bs}^{m,t})+\frac{1}{\delta_{PA}^{m}}P_{tr}^{m,t}+P_{AAU,Ac}^{m,c}$$

(19) $$P_{AAU,Sl}^{m,t}\geq -M\cdot U_{bs}^{m,t}+P_{AAU,Sl}^{m,c}$$

(20) $$P_{AAU,Ac}^{m,t},P_{AAU,Sl}^{m,t}\geq 0$$

(21) $$P_{BBU}^{m,t}=P_{BBU,Ac}^{m,t}+P_{BBU,Sl}^{m,t}$$

(22) $$P_{BBU,Ac}^{m,t}\geq -M(1-U_{bs}^{m,t})+P_{BBU,Ac}^{m,c}$$

(23) $$P_{BBU,Sl}^{m,t}\geq -M\cdot U_{bs}^{m,t}+P_{BBU,Sl}^{m,c}$$

(24) $$P_{BBU,Ac}^{m,t},P_{BBU,Sl}^{m,t}\geq 0$$

where $P_{AAU,Ac}^{m,t}$ and $P_{AAU,Sl}^{m,t}$ are the powers of AAU *m* when BS *m* is in active and sleep mode, respectively; $P_{BBU,Ac}^{m,t}$ and $P_{BBU,Sl}^{m,t}$ are the powers of BBU *m* when BS *m* is in active and sleep mode, respectively; and *M* is a sufficiently large number.

In summary, the optimization of the energy management of communications equipment in the 5G macro Bs network is shown as ($P_{1}^{t}$).

$$\begin{array}{l} (P_{1}^{t})\min F_{1}^{t}=\min\sum_{m\in M}P_{com}^{m,t}\Delta t\\ s.t.(2),(5),(7),(11)-(24) \end{array}$$

It can be observed that ($P_{1}^{t}$) is a single-step optimization problem. Solving ($P_{1}^{t}$) for each time step t∈T, we obtain the optimal on/off state $\left\{{\hat{U}}_{bs}^{m,t}\right\}$, user association method $\left\{{\hat{a}}_{t}^{m,m_{i}^{\prime }}\right\}$, and power profile $\left\{P_{com}^{m,t^{*}}\right\}$ of the communications equipment in each 5G BS.

### 2.3. Optimization Step 2: Energy Management of the Standard Equipment in 5G Macro BSs Network

The energy management model of communications equipment in the 5G macro BS network was described in the previous section. BS sleeping and user allocation strategies were adopted to minimize the energy consumption of the communication equipment. However, ACs, backup batteries, and renewable generation units can be optimized to further reduce the electricity costs of 5G networks.

In this section, the operation of ACs, backup batteries, and renewable generation units are jointly optimized under the given power consumption profile of the communications equipment. Two-way energy trading between the 5G network and the grid was not considered in this study. The objective is to minimize the electricity costs of the entire 5G macro BS network, as shown in Equation (25).

(25) $$\min F_{2}=\min\sum_{t\in T}c_{t}P_{grid}^{t}\Delta t$$

where *c_t_* denotes the electricity price, and $P_{grid}^{t}$ represents the input power to the 5G macro BS network from the grid.

The operation of the above equipment needs to meet the following constraints.

(1) Power balance constraints of 5G macro BSs network:
  (26) $$P_{grid}^{t}=\sum_{m\in M}P_{in}^{m,t}$$
  (27) $$P_{grid}^{t}\geq 0$$
  where $P_{in}^{m,t}$ represents the input power of BS *m*, which can be either positive or negative.
(2) Power balance constraints of a single BS:
  (28) $$P_{in}^{m,t}+P_{dis}^{m,t}+(1-Cur_{t}^{m})P_{RE}^{m,t*}=P_{air}^{m,t}+P_{ch}^{m,t}+P_{com}^{m,t*}$$
  where $P_{ch}^{m,t}$, $P_{dis}^{m,t}$, and $P_{air}^{m,t}$ are the charging/discharging power of the backup battery and power of the AC in BS *m*; ${Cur}_{t}^{m}$ denotes the wind/solar curtailment rate of the renewable generation unit in BS *m*; and $P_{RE}^{m,t^{*}}$ and $P_{com}^{m,t^{*}}$ are the given power profiles of the renewable generation unit and communication equipment in BS *m*, respectively.
(3) AC-related constraints:
  (29) $$P_{air}^{m,t}=P_{air}^{m,N}U_{air}^{m,t}$$
  (30) $$P_{cool}^{m,t}=\delta^{air}P_{air}^{m,t}-\delta^{hot}(P_{BBU,Ac}^{m,c}U_{bs}^{m,t*}+P_{other})$$
  (31) $$T_{in}^{m,t}=T_{in}^{m,init},t=1$$
  (32) $$T_{in}^{m,t}=T_{out}^{t-1}-R_{eq}^{m}P_{cool}^{m,t}-(T_{out}^{t-1}-R_{eq}^{m}P_{cool}^{m,t}-T_{in}^{m,t-1})e^{-\frac{\Delta t}{R_{eq}^{m}C_{eq}^{m}}},t=2,3,\ldots ,T+1$$
  (33) $$T_{in}^{\min}\leq T_{in}^{m,t}\leq T_{in}^{\max},t=1,2,\ldots ,T+1$$
  (34) $$U_{air}^{m,t}\in \{0,1\}$$
  where $P_{air}^{m,N}$ is the rated power of the fixed frequency of AC in BS *m*; $U_{air}^{m,t}$ denotes the on/off state of AC; $P_{cool}^{m,t}$ represents the equivalent cooling power inside BS m; $\delta^{air}$ is the energy efficiency ratio of AC in BS *m*; and *P_other_* represents the heating power of the other equipment in the cabinet of BS *m*. $T_{in}^{m,t}$ represents the indoor temperature; $T_{in}^{m,init}$ denotes the initial indoor temperature in the cabinet of BS *m* when *t* = 1, $T_{in}^{max}$, and $T_{in}^{min}$ are the preset upper and lower bounds of the indoor temperature inside the cabinet, respectively; and $R_{eq}^{m}$ and $C_{eq}^{m}$ denote the equivalent thermal resistance and equivalent thermal capacity of the cabinet of BS *m*, respectively.
(4) Backup battery-related constraints:
  (35) $$P_{ch}^{\min}U_{ch}^{m,t}\leq P_{ch}^{m,t}\leq P_{ch}^{\max}U_{ch}^{m,t}$$
  (36) $$P_{dis}^{\min}U_{dis}^{m,t}\leq P_{dis}^{m,t}\leq P_{dis}^{\max}U_{dis}^{m,t}$$
  (37) $$C_{bat}^{m,t}=C_{bat}^{m,init},t=1$$
  (38) $$C_{bat}^{m,t}=\xi^{leak}C_{bat}^{m,t-1}+\delta^{bat}P_{ch}^{m,t}\Delta t-\frac{1}{\delta^{bat}}P_{dis}^{m,t}\Delta t,t=2,3,\ldots ,T+1$$
  (39) $$C_{bat}^{\min}\leq C_{bat}^{m,t}\leq C_{bat}^{\max}$$
  (40) $$0\leq U_{ch}^{m,t}+U_{dis}^{m,t}\leq 1$$
  (41) $$U_{ch}^{m,t},U_{dis}^{m,t}\in \{0,1\}$$
  where $U_{ch}^{m,t}$ and $U_{dis}^{m,t}$ denote the charge/discharge state of the backup battery in BS *m*; $P_{ch}^{min}$, $P_{ch}^{max}$, $P_{dis}^{min}$, and $P_{dis}^{max}$ are the upper/lower limits of the charging power and discharging power of the backup battery in BS *m*; $C_{bat}^{m,t}$ denotes the energy storage of the backup battery in BS m; $C_{bat}^{m,init}$ denotes the initial energy storage of the backup battery in BS m; $\xi^{leak}$ denotes the energy leakage coefficient of the battery; $\delta^{bat}$ is the charge/discharge efficiency coefficient of the battery; and $C_{bat}^{max}$ and $C_{bat}^{min}$ represent the upper and lower limits of the energy storage of the backup battery in each BS, respectively.
(5) Wind/solar curtailment rate constraints:
  (42) $$0\leq Cur_{t}^{m}\leq 1$$

In summary, the problem of jointly optimizing the operation of ACs, storage batteries, and renewable generation units is denoted as

$$\begin{array}{l} (P_{2})\min F_{2}=\min\sum_{t\in T}c_{t}P_{grid}^{t}\Delta t\\ s.t.(26)-(42) \end{array}$$

## 3. Problem Reformulation with User Clustering and Benders Decomposition

Compared with 4G, the number of 5G terminal devices has grown explosively. If we consider the optimal allocation of each user in ($P_{1}^{t}$), the optimization problem will be too complex to be solved. In addition, (**P**_2_) is a large-scale, mixed-integer linear programming problem (MILP) and is difficult to solve directly when the number of considered BSs grows. To solve the above problems, a user clustering method for ($P_{1}^{t}$) and the Benders decomposition algorithm for (**P**_2_) are adopted, respectively, to accelerate the solution process.

### 3.1. User Clustering Strategy for ($P_{1}^{t}$)

**Figure 4:**
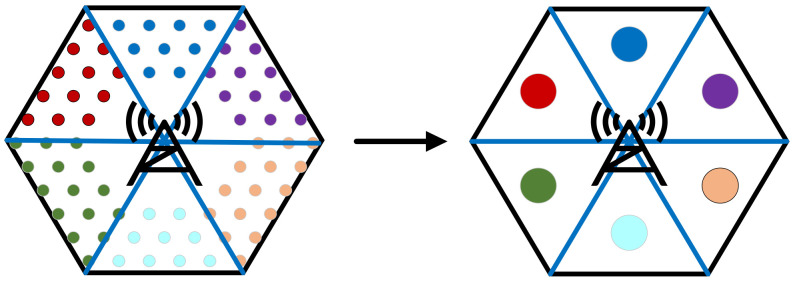
Schematic of the user clustering method.

To solve the problem in which the scale of ($P_{1}^{t}$) grows with the number of users, user clustering is adopted in this work. Each cell *m* is evenly divided into six sub-cells *m_i_* (*i* = 1, 2,…, 6), as shown in Figure 4. All users in the same sub-cell *m_i_* belong to user cluster *m_i_* and are deployed as a whole. In this way, the number of binary variables in ($P_{1}^{t}$) is greatly reduced and does not increase with an increase in the number of users in the network.

After the user clustering strategy is adopted, all users in cluster *m_i_’* may be connected to one of the $\left|N_{m_{i}^{\prime }}\right|$ adjacent BSs.

The data rate from BS *m* to cluster *m_i_′* is shown in Equation (43) when they are connected.

(43) $$L_{tr,t}^{m,m_{i}\prime }=\sum_{u_{t}\in U_{m_{i}\prime }}L_{re}^{u_{t}}$$

where $L_{tr,t}^{m,m_{i}^{\prime }}$ denotes the data rate from BS *m* to cluster *m_i_’*, and $U_{m_{i}^{\prime }}$ denotes the set of users in cluster *m_i_′*.

Meanwhile, BS *m* needs to allocate transmit power $P_{tr,t}^{m,m_{i}^{\prime }}$ to cluster *m_i_’* to meet user needs, as shown in Equation (44).

(44) $$P_{tr,t}^{m,m_{i}\prime }=\sum_{u_{t}\in U_{m_{i}\prime }}P_{tr}^{m,u_{t}}$$

where $P_{tr,t}^{m,m_{i}^{\prime }}$ denotes the transmit power from BS *m* to cluster *m_i_’*.

The constraints expressed by Equations (11)–(16) are converted to Equations (46)–(51).

(45) $$a_{t}^{m,m_{i}\prime }\in \{0,1\}$$

(46) $$a_{t}^{m,m_{i}\prime }\leq U_{bs}^{m,t},m\in N_{m_{i}\prime }$$

(47) $$\sum_{m\in N_{m_{i}\prime }}a_{t}^{m,m_{i}\prime }=1$$

(48) $$L_{tr}^{m,t}=\sum_{m_{i}\prime \in N_{m}^{cl}}a_{t}^{m,m_{i}\prime }L_{tr,t}^{m,m_{i}\prime }$$

(49) $$L_{tr}^{m,t}\leq L_{tr}^{\max,m}$$

(50) $$P_{tr}^{m,t}=\sum_{m_{i}\prime \in N_{m}^{cl}}a_{t}^{m,m_{i}\prime }P_{tr,t}^{m,m_{i}\prime }$$

(51) $$P_{tr}^{m,t}\leq P_{tr}^{\max,m}$$

where $a_{t}^{m,m_{i}^{\prime }}$ denotes the connection relationship between BS *m* and cluster *m_i_’*; $N_{m_{i}^{\prime }}$ denotes the set of adjacent BSs of cluster *m_i_’*; and $N_{m}^{cl}$ denotes the set of clusters in cell *m* and its adjacent cells.

Thus, ($P_{1}^{t}$) is shown as follows.

$$\begin{array}{l} (P_{1}^{t})\min F_{1}^{t}=\min\sum_{m\in M}P_{com}^{m,t}\Delta t\\ s.t.(2),(5),(7),(10),(17)-(24),(43)-(51) \end{array}$$

The proposed user clustering method according to user locations has obvious practical value. According to Equation (3), the transmit power needed by user *u* is positively correlated with the distance between *u* and its connected BS *m*. It is easy to observe that the set of adjacent BSs ***N****_u_* of each user *u* in cluster *m_i_’* is the same. The size relationship of the geographic distance between user *u* and each BS *m* (*m* ∈ ***N****_u_*) is basically uniform. Therefore, the size relationship of the required transmitting power of each user *u* in the same cluster *m_i_’* is basically uniform when connected to each BS *m* (*m* ∈ ***N****_u_*). This property ensures that the optimal solution of ($P_{1}^{t}$) with or without the user clustering strategy is similar, while considerable calculation time is saved.

### 3.2. Benders Decomposition for (P_2_)

Because the continuity equations for indoor temperature (Equation (32)) and backup batteries (Equation (38)) are both coupling constraints between adjacent time steps, (**P**_2_) is a large-scale MILP of a multi-device, multi-step, jointly optimal scheduling, which cannot be decomposed into |***T***| single-step optimality problems in the form of ($P_{1}^{t}$) and is difficult to solve directly.

**Figure 5:**
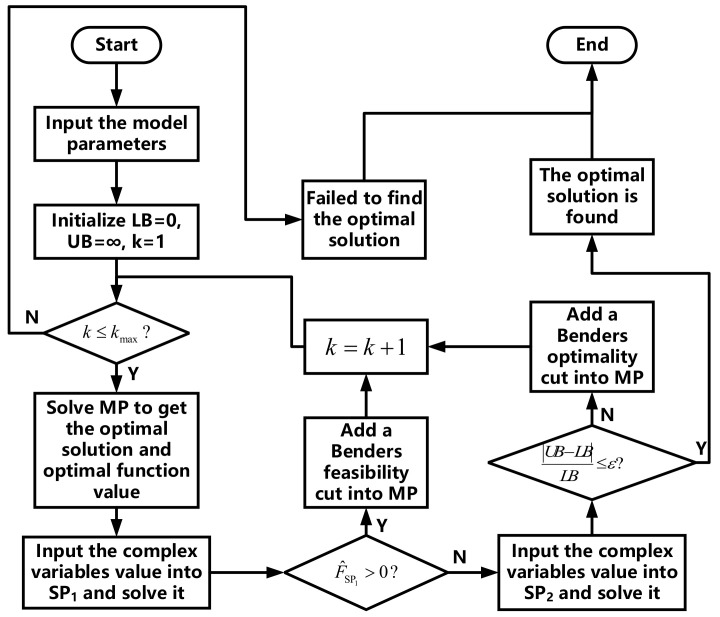
Flow chart of Benders decomposition for application to (**P**_2_).

The Benders decomposition algorithm [35] is extensively applied to decompose large-scale MILPs. By selecting complex variables from all variables in (**P**_2_), the problem is decomposed to a master problem (MP), a feasibility subproblem (SP_1_), and an optimality subproblem (SP_2_) to decrease the complexity of the original problem. In this study, all integer variables ($\left\{U_{air}^{m,t}\right\}$, $\left\{U_{ch}^{m,t}\right\}$, and $\left\{U_{dis}^{m,t}\right\}$), and all AC-related continuous variables ($\left\{P_{air}^{m,t}\right\}$, $\left\{P_{cool}^{m,t}\right\}$, and $\left\{T_{in}^{m,t}\right\}$) in (**P**_2_) are included in the MP, and the other continuous variables are included in the SPs. The flow chart of the Benders decomposition algorithm applied to (**P**_2_) is shown in Figure 5. The specific application steps are as follows:

(1) Input the model parameters, initialize the lower and upper bounds of (**P**_2_) *LB* = 0 and *UB* = ∞, and initialize the number of iterations *k* = 1;
(2) Check whether the condition pertaining to the number of iterations *k* ≤ *k_max_* is established. If it is, then go to step (3); if it is not, then the loop ends, and the optimal solution of (**P**_2_) fails to be found in *k_max_* iterations;
(3) Solve the MP to obtain the optimal function value $\hat{\theta }$ and optimal variable values ($\left\{{\hat{U}}_{air}^{m,t}\right\}$, $\left\{{\hat{U}}_{ch}^{m,t}\right\}$, $\left\{{\hat{U}}_{dis}^{m,t}\right\}$, $\left\{{\hat{P}}_{cool}^{m,t}\right\}$, and $\left\{{\hat{T}}_{in}^{m,t}\right\}$; let $LB=\hat{\theta }$;
  Input the value of the complex variables into SP_1_ and then solve the optimal function value ${\hat{F}}_{SP_{1}}$ of SP_1_ and check whether ${\hat{F}}_{SP_{1}}\geq 0$ is established; if it is, then add a Benders feasibility cut into the MP, let *k = k +* 1; if it is not, then go to step (5);
(5) Input the value of the complex variables into SP_2_, and then solve the optimal function value ${\hat{F}}_{SP_{2}}$ of SP_2_; let $UB={\hat{F}}_{SP_{2}}$;
(6) Check whether the convergence criterion $\left|UB-LB\right|/LB\leq \varepsilon$ is established. If it is, then the optimal solution of (**P**_2_) is found, and the loop ends; if it is not, add a Benders optimality cut to MP, let *k = k +* 1, and go to step (2).

## 4. Case Study

In this section, numerical results are presented to validate the effectiveness of the proposed two-step energy management model and the corresponding accelerating solution method. Two cases are studied: one is a 3 × 3 5G macro BS network, and the other is a 10 × 10 5G macro BS network.

The simulation is based on MATLAB R2018a (MathWorks, Natick, MA, USA) with an Intel Core i5-10400F at 2.90 GHz and 24 GB random access memory (RAM). A Gurobi 9.1.1 commercial solver was used to solve the model.

### 4.1. Simulation on 3 × 3 5G Macro BSs Network

#### 4.1.1. System Description

**Figure 6:**
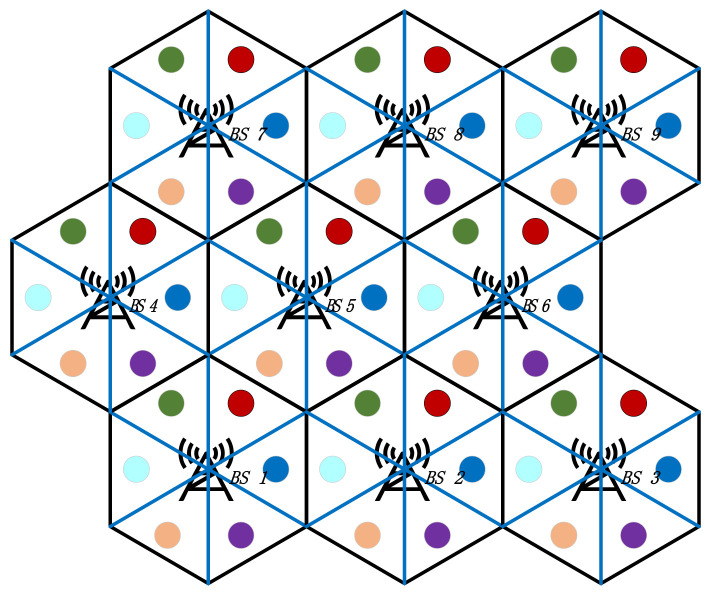
Diagram of a 3 × 3 distributed 5G macro BS network.

The simulated 3 × 3 5G macro BS network is shown in Figure 6 with the geographic distribution of each cell. The dot in each subcell represents the cluster of all users in that subcell. The traffic load, renewable generation power, and electricity price profiles are shown in Appendix A.

#### 4.1.2. Effectiveness of the Two-Step Energy Management Model for 5G Macro BS Network

First, the performance of the proposed two-step energy management model for the 5G macro BS network for the improvement of the dispatching scheme of both communication and standard equipment is evaluated.

**Figure 7:**
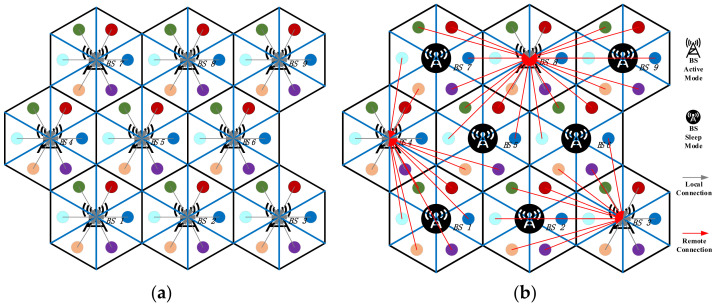
On/off states of BSs and cluster association strategy: (**a**) peak period of traffic load and (**b**) valley period of traffic load.

The dispatching scheme of the communication equipment and users with the use of the proposed model is analyzed first. The on/off state and cluster association strategy during the period of peak/valley traffic load (t_peak_ = 73, t_valley_ = 18) are shown in Figure 7a,b, respectively.

It can be observed from Figure 7a that during the period of peak traffic load, all of the BSs are heavily occupied; hence, the users are unable to transfer to adjacent cells. All BSs in the 5G network are in active mode, and the users in each cell are served by the local BSs. According to Figure 7b, during the period of valley traffic load, some BSs in the network are lightly loaded so that the redundant BSs (BS 1, 2, 5, 6, 7, and 9) are switched to the sleep mode and the users in the corresponding cells are transferred to adjacent BSs to receive communication services. With the use of the BS sleeping strategy and user transferring strategy, the 5G macro BSs in the network coordinate with each other to reduce electricity costs and energy consumption. It can be observed that with the proposed dispatching scheme, the energy consumption of the communication equipment is reduced by making full use of the spatial and temporal fluctuations of the traffic load.

**Figure 8:**
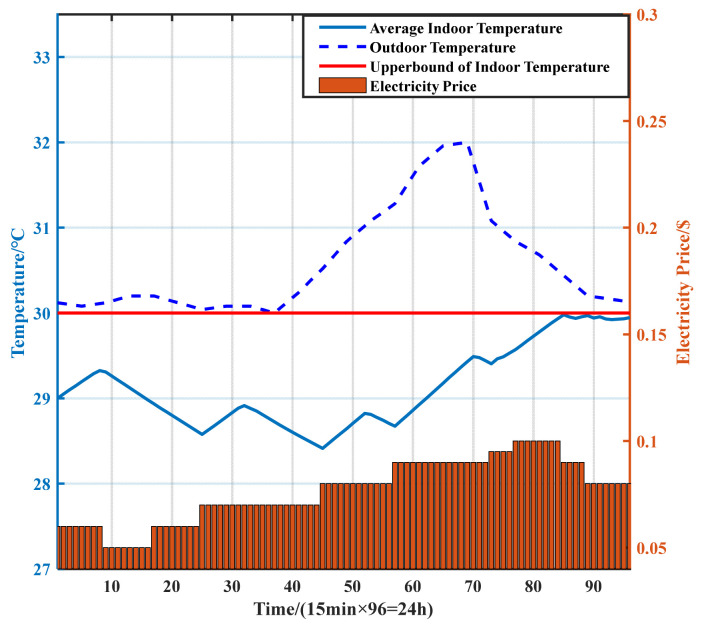
Average indoor temperature.

The average indoor temperatures are shown in Figure 8. To ensure the normal working status of the BSs, the AC needs to maintain the temperature inside the cabinets below the upper bound of the indoor temperature. The thermal inertia of the cabinet can be used to shift the power consumption of the air conditioner. When the electricity price is relatively low, such as in periods 9–24, the AC is turned on to reduce the indoor temperature to a low level, while in instances in which the electricity price is high, such as in periods 25–31, the AC can be turned off. The indoor temperature would not reach the upper bound of the indoor temperature owing to the thermal inertia of the cabinets.

In addition, during periods in which the electricity prices and outdoor temperature are both high (i.e., periods 45–96), the high-cooling demand and electricity prices will lead to high-electricity costs produced by the AC. In this regard, with the proposed dispatching scheme, the AC is first turned on in advance (during periods 32–44, when the electricity price and outdoor temperature are relatively low) to reduce the indoor temperature to a low level so that the working time of the AC can be shortened during the following periods. The power consumption of AC is as conservative as possible during periods with a high electricity price and outdoor temperature. In detail, the overall trend of indoor temperature is rising, and the AC is turned on for a short time during periods 52–56 and 70–72 to keep the indoor temperature below the upper bound. After period 85, the indoor temperature reached its upper limit. In these periods, the AC only turned on when the indoor temperature almost exceeded the upper bound instead of lowering the indoor temperature so that the power consumption and electricity costs could be reduced.

**Figure 9:**
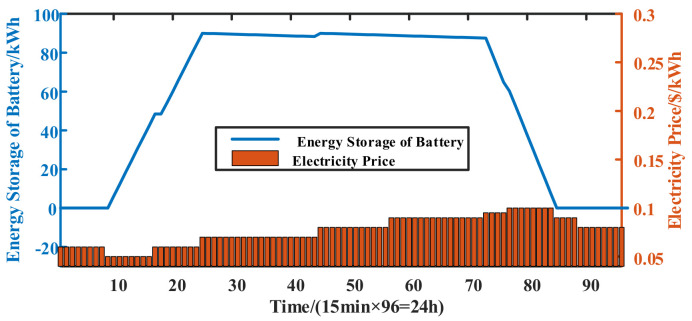
Energy storage of backup batteries in the 5G macro BS network.

The variation in the total energy storage of backup batteries in the 5G network with the electricity price is shown in Figure 9. As shown, the charging action mainly occurs during the low electricity price periods (i.e., periods 10–25), and the discharging action mainly occurs during the high electricity price periods (i.e., periods 75–85). The low-priced electric energy is transferred to the high price period, thus reducing the total power electricity costs of the network.

In summary, with the proposed dispatching scheme, the power consumption and electricity costs of the 5G macro BS network can be reduced by taking advantage of the spatial and temporal fluctuations of the traffic load, the thermal inertia of the cabinets, and the storage of the backup batteries.

#### 4.1.3. Computational Efficiency of the User Clustering Method and Benders Decomposition

To demonstrate the computational efficiency of the proposed user clustering method and Benders decomposition, the following three cases were tested for comparison.

Case 1: In the original model proposed in Section 2 for the 5G macro BS network, neither the proposed user clustering methods and Benders decomposition were applied.

Case 2: In the two-step energy management model for the 5G macro BS network, the proposed user clustering method was applied to reduce the computational burden of the first-step problem. Benders decomposition was not applied to the second step problem.

Case 3: In the reformulated model proposed in Section 3 for the 5G macro BS network, both the proposed user clustering method and Benders decomposition were applied to the first and the second step problems, respectively.

Table 1 lists the comparative results for the three cases.

Based on the comparison between Cases 1 and 2 in Table 1, the objective values of (**P**_1_) and (**P**_2_) based on the use of the proposed user clustering method (Case 2) are 0.442% and 0.436% larger than that of Case 1, respectively, validating the achievement of approximately optimal performance. In addition, the user clustering method (Case 2) outperforms Case 1 in terms of calculation time because it can reduce a large number of integer variables, and the solving time of (**P**_1_) is reduced by approximately 80%. However, the long calculation time of (**P**_2_) remains a problem that needs to be solved.

By comparing Cases 2 and 3 in Table 1, the reformulated model (Case 3) combines the advantages of the user clustering method and Benders decomposition. The objective values of (**P**_1_) and (**P**_2_) of Case 3 are the same as those of Case 2, which is also shown to reach approximately optimal performance. It can also be observed that the application of Benders decomposition does not reduce optimality. In addition, the reformulated model (Case 3) outperforms the other two cases in terms of the total calculation time because it reduces the number of integer variables in (**P**_1_) and the size of (**P**_2_) by the decomposition method.

In summary, with the proposed user clustering method and Benders decomposition, the proposed dispatching scheme can achieve approximately optimal performance, while the solution time is reduced considerably.

### 4.2. Simulation on 10 × 10 5G Macro BS Network

#### 4.2.1. System Description

The simulation was conducted on a 10 × 10 5G macro BS network. The traffic load profile and renewable generation power of the network are presented in Appendix A.

#### 4.2.2. Comparative Analysis between the Proposed Dispatching Scheme and the Conventional Dispatching Scheme for 5G Macro BSs Network

To verify the performance of the proposed two-step energy management model in terms of saving energy consumption and electricity costs, the following two dispatching schemes were tested for comparison.

Dispatching scheme 1 (DS 1): The dispatching scheme of communication and standard equipment in the 5G macro BS network is determined by the proposed two-step energy management model proposed in Section 3.

Dispatching scheme 2 (DS 2): The conventional dispatching scheme. All BSs in the network are always in active mode, and the users in each cell are served by the 5G macro BS in the local cell; that is, user allocation is not performed, the transmission of electric energy among the BSs is not performed, the fixed-frequency commercial AC is temperature-controlled, and the set temperature is fixed. When the indoor temperature exceeds the setting upper value, the AC turns on, and it turns off when the indoor temperature exceeds the setting lower value. The backup batteries are used as a UPS, and they do not charge and discharge in normal conditions. The power generation of renewable energy is consumed by the local BS, and excess renewable energy is curtailed.

Table 2 presents the comparative results of the two schemes. It can be observed that with DS 1, the electricity cost of the network is 20.35% less than that of DS 2. The energy consumption of the network with DS 1 is 15.90% higher than that of DS 2. The energy consumptions of the communication equipment and AC are both reduced with DS 1. The analysis outcomes are summarized in Table 2.

**Figure 10:**
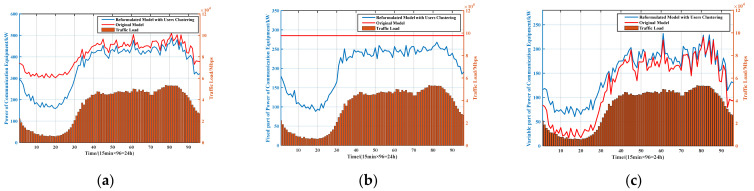
(**a**) Total power of communication equipment, (**b**) fixed part of the power of communication equipment, and (**c**) variable part of the power of communication equipment.

First, we compared the dispatching scheme of the communication equipment based on the use of two strategies. The total power, fixed part of the power, and variable part of the power of the communication equipment are shown in Figure 10a–c, respectively.

It can be observed from Figure 10a that the power variations of the communication equipment in the network with DS 1 and 2 are basically consistent with the traffic load profile. The power of the communication equipment with DS 1 is always lower than that of DS 2. The power of communication equipment with the above two strategies is close during high-traffic periods (such as the periods 40–85) and quite different during low-traffic periods (such as periods 10–30).

According to Equation (1), the power of communication equipment in active mode is composed of a fixed and a variable power, in which the variable power is proportional to the BSs’ transmission power. As shown in Figure 10b, with DS 2, the communication equipment is always in active mode, meaning that the fixed power has been maintained at the maximum value; with DS 1, the redundant BSs can be shut down during the low-traffic period, and the sleeping BSs are “awakened” in high-traffic periods to serve the users, thereby reducing the fixed power consumption of communication equipment. According to Figure 10c, the variable power of the communication equipment with DS 1 is higher than that of DS 2. This is because, with DS 1, after the redundant BSs switch to sleep mode, the users in the corresponding cells are transferred to adjacent BSs to receive communication services. The transfer of users increases the geographic distance between the users and BSs. According to Equation (5), an increase in distance leads to an increase in variable power. However, in general, with DS 1, the reduction in fixed power is greater than the increase in variable power, and thus the purpose of reducing the energy consumption and the electricity costs can be achieved.

**Figure 11:**
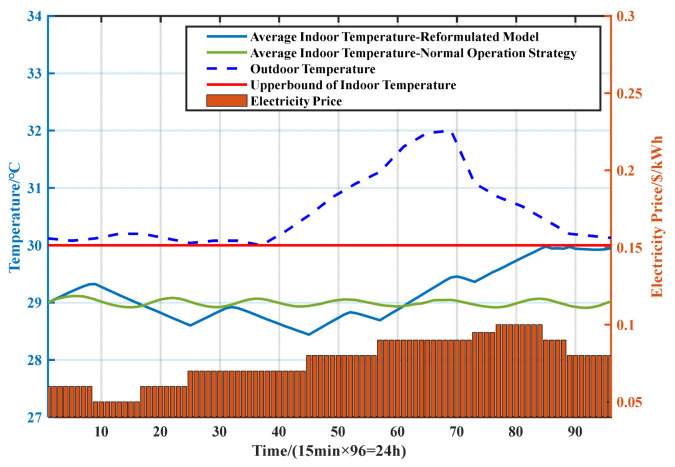
Average indoor temperature.

We then compared the dispatching scheme of AC with two strategies, and the average indoor temperature in the cabinets is shown in Figure 11. The analysis of the dispatching scheme of AC with DS 1 is consistent with that in Section 4.1.2. With DS 2, the dispatching scheme of AC only depends on whether the indoor temperature exceeds the preset temperature range. According to Figure 11, during periods in which the outdoor temperature and electricity prices are both high, the working time of AC with DS 2 is much longer than that of DS 1. In addition, the AC fails to reduce the indoor temperature in advance before the outdoor temperature and electricity price increase; in other words, the AC fails to make full use of the thermal inertia of the cabinets, thus resulting in higher electricity costs. Furthermore, the indoor temperature with DS 2 can only be maintained within the preset temperature range, whereas the proposed DS 1 allows the indoor temperature to approach the upper bound. This is an important reason for the lower energy consumption and electricity costs with DS 1 compared with DS 2.

**Figure 12:**
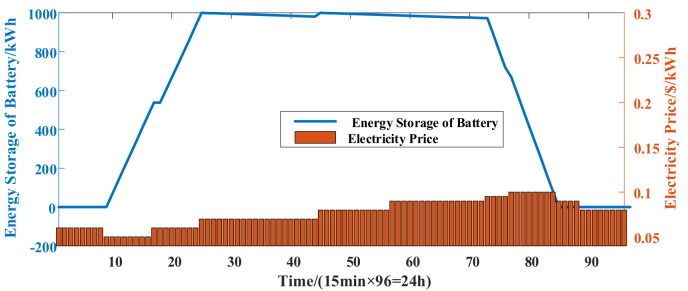
Energy storage of backup batteries in the 5G macro BS network.

Finally, the backup batteries are allowed to charge and discharge with DS 1. The total energy storage of backup batteries in the 5G macro BS network is shown in Figure 12. The analysis of the dispatching scheme of batteries is consistent with that described in Section 4.1.2. By storing and releasing power from the grid over time, the power during the low electricity price period is transferred to the period with a high electricity price. Compared with DS 2, where the batteries are not allowed to be dispatched, the proposed DS 1 effectively reduces the electricity costs of the network.

In summary, with the proposed dispatching scheme in this study, through the coordination of each 5G BS and the economic dispatch of equipment in the network, the energy consumption and electricity costs are effectively reduced in comparison with the conventional dispatching scheme.

## 5. Conclusions

In this study, a two-step optimal energy management for a 5G macro BS network was developed to coordinate the BSs’ on/off states, user allocation, and power transmission among BSs in the network. A user clustering method based on geographic location was proposed to reduce the computational burden, and the Benders decomposition method was applied to accelerate problem solving. Case studies were conducted on a 3 × 3 5G macro BS network and a 10 × 10 5G macro BS network. The key findings of the simulation results are summarized as follows:

- The two-step energy management model for communication and standard equipment can effectively reduce the energy consumption and electricity costs of the entire 5G macro BS network compared with the conventional dispatching scheme by making full use of the spatial and temporal fluctuations of the traffic load, the thermal inertia of the cabinets, and the storage of the backup batteries;
- The proposed solution-accelerating methods—that is, user clustering and Benders decomposition—were found to be computationally efficient, while they ensured excellent performance with approximate optimality.

## Figures and Tables

**Table 1:** Comparative results for the three tested cases.

Case	Objective Value of (P_1_) (kWh)	Optimality Gap	Calculation Time (s)	Objective Value of (P_2_) ($)	Optimality Gap	Calculation Time (s)
Case 1	588.3	0	5.3518	48.21	0	582.7
Case 2	590.9	0.442%	1.1317	48.42	0.436%	469.5
Case 3	590.9	0.442%	0.7798	48.42	0.436%	40.3

**Table 2:** Comparison of the simulation results of DS 1 and DS 2.

Operation Strategy	Cost/$	E_grid_/kWh	E_com_/kWh	E_air_/kWh
DS 1	693.82	9402.29	8431.56	1651.50
DS 2	871.11	11,179.75	9827.16	2305.50

Where Cost represents the total electricity costs of the 5G macro BS network, Egrid represents the total energy consumption from the grid of the network, and Ecom and Eair are the total energy consumption of communication equipment and AC in the network, respectively.

**Figure A1:**
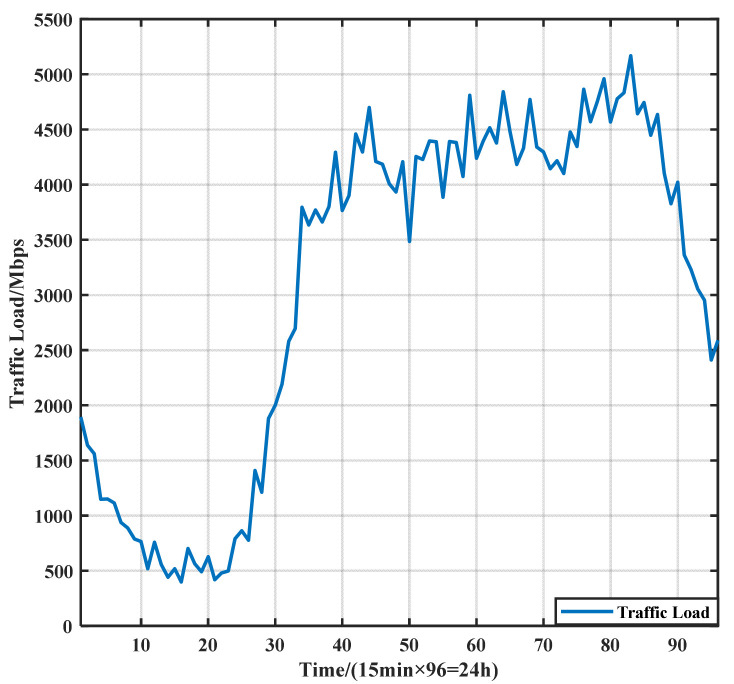
Traffic load of a 3 × 3 fifth generation mobile communications technology (5G) macro base station (BS) network.

**Figure A2:**
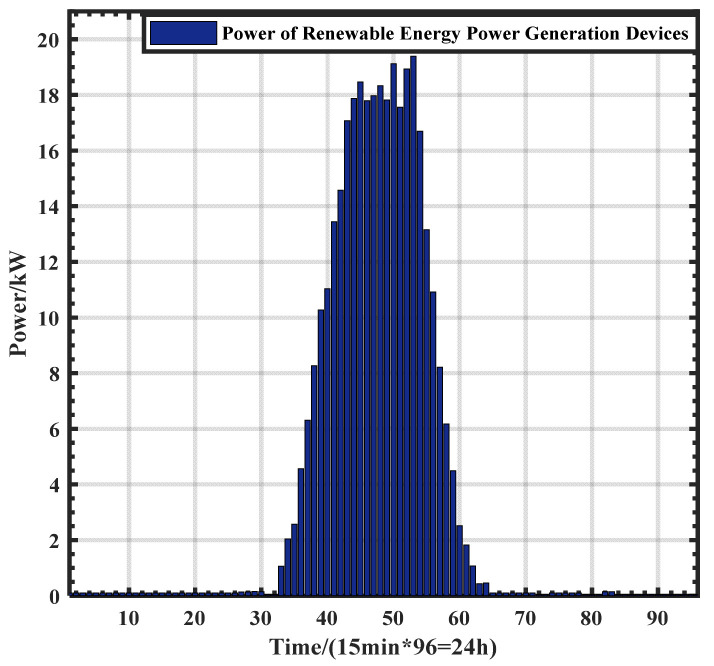
Power generation of renewable generation units of a 3 × 3 5G macro BS network.

**Figure A3:**
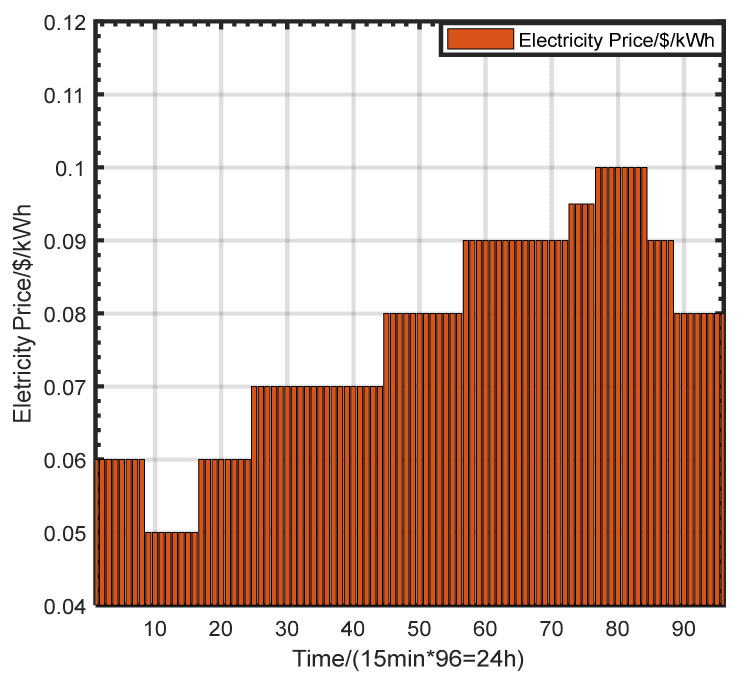
Electricity price.

**Figure A4:**
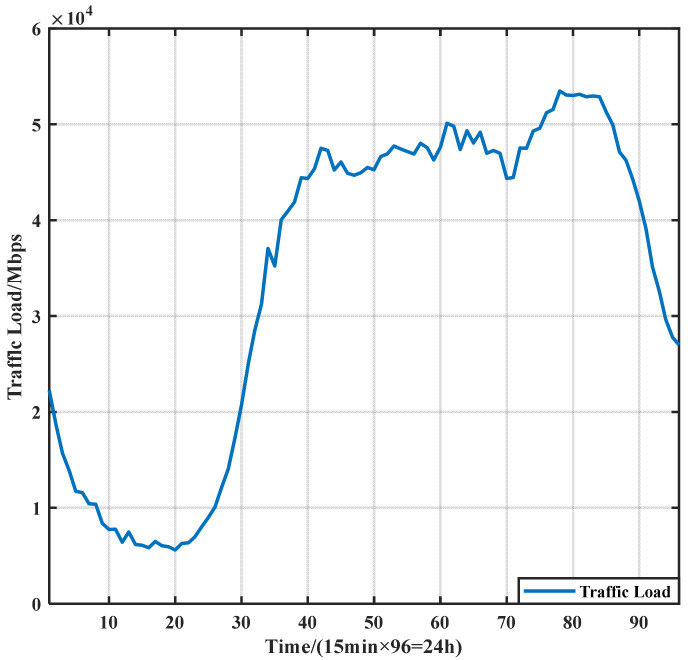
Traffic load of a 10 × 10 5G macro BS network.

**Figure A5:**
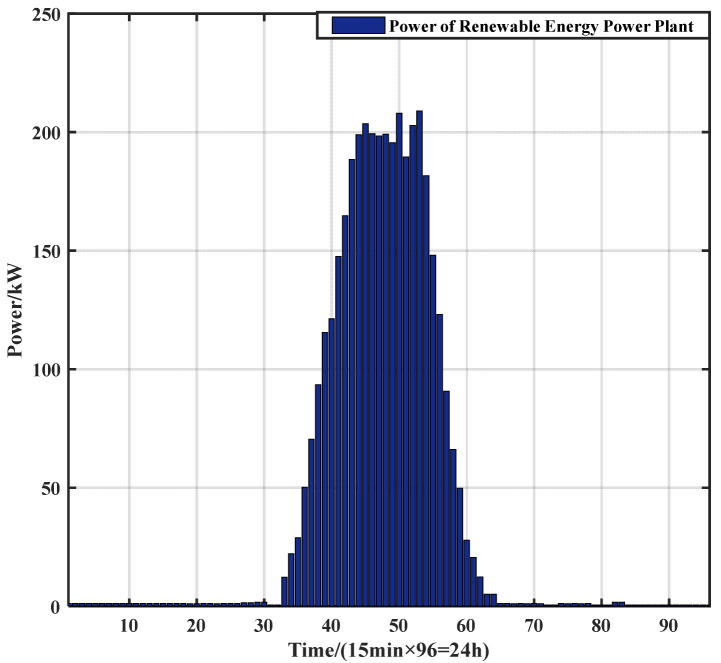
Power generation of renewable generation units of a 10 × 10 5G macro BS network.

## Data Availability

Data sharing is not applicable to this article.

## Appendix A

**A 3 × 3 5G macro BS network.**

**A 10 × 10 5G macro BS network**.
